# Cerebral vein thrombosis in a woman using oral contraceptive pills for a short period of time: a case report

**DOI:** 10.1186/s13256-022-03473-w

**Published:** 2022-07-04

**Authors:** Somayeh Moeindarbari, Nazanin Beheshtian, Shima Hashemi

**Affiliations:** 1 Department of Obstetrics and Gynecology, Neonatal and Maternal Research Center, Mashhad University of Medical Sciences, Mashhad, Iran; 2 Department of Obstetrics and Gynecology, School of Medicine, Imam Reza Hospital, Mashhad University of Medical Sciences, Mashhad, Iran; 3 Department of Epidemiology, Faculty of Health, Ilam University of Medical Sciences, Ilam, Iran

**Keywords:** Cerebral vein thrombosis, Oral contraceptive pill, OCP, Woman

## Abstract

**Background:**

Cerebral vein thrombosis is increasing in young adults. Although oral contraceptive pills increase the risk of cerebral vein thrombosis, relatively high brain venous involvement is rare when oral contraceptive pills are consumed for a short duration.

**Case presentation:**

A 31-year-old Asian woman was referred to Imam Reza Hospital with a headache complaint on 11 November 2020. The woman, who had a headache for the previous 11 days, went to the hospital. Owing to endometriosis involvement, she consumed Diane tablets. According to the imaging findings, three vein involvements were diagnosed. Anticoagulant therapy was started, and the symptoms disappeared.

**Conclusions:**

All cerebral vein thrombosis symptoms are variable, but new presentation of headache could be an early symptom of cerebral vein thrombosis.

## Background

According to epidemiological studies, the prevalence rate of cerebral vein thrombosis (CVT) is 10–12 per million in Iran and 4–7 per million in the world [1, 2]. One or more sinuses and cerebral veins are blocked, reducing the blood flow of the brain and cerebrospinal fluid [2]. CVT clinical manifestation depends on thrombosis location and includes: headache, seizure, focal neurological deficit, increasing intracranial pressure, loss of consciousness, vertigo, and vomiting [1, 3, 4]. CVT increases in young adults, women of childbearing age, and children. Affected children are usually newborns. The age of patients with CVT ranges from newborn to 82 years old, but this condition occurs mainly in individuals between 30 and 41 years old [3]. Although the mortality rate of CVT has been significantly reduced by improvements in treatment and diagnostic techniques, the mortality rate of severe CVT remains as high as 34.2%. Considering the risk factors can help to diagnose it. The incidence of infectious CVT was significantly reduced by the use of antibiotics, whereas the incidence of CVT associated with other factors, including trauma, Behcet’s disease, the perinatal period, use of oral contraceptives, neoplasms, nephrotic syndrome, coagulation factor, and other abnormalities, was higher. With the advent of new diagnostic techniques, such as computed tomographic venography (CTV) and magnetic resonance angiography (MRA), it has become easier to achieve an early diagnosis of CVT [5]. Because of the comfortable use of oral contraceptive pills (OCPs), their daily use is increasing among women [5]. OCPs are known as an effective contraception and are prescribed for the treatment of some diseases. Oral contraceptives seem to increase the risk of cerebral venous sinus thrombosis (CVST) owing to their estrogenic component because estrogens increase the levels of coagulation factors and decrease the levels of anticoagulant proteins such as antithrombin and proteins. According to several observational studies, females who consumed OCPs had a 5- to 22-fold-increased risk of CVT. Among the various types of combined hormonal contraceptives (CHCs), third-generation CHCs containing desogestrel or gestoden reportedly confer the highest risk for CVT [6, 7]. Although the effect of OCP increases the risk of CVT, relatively high brain venous involvement is rare in short-duration consumption of OCPs.

## Case presentation

A 31-year-old Asian woman was referred to Imam Reza Hospital with headache complaint lasting 11 days in all parts of the head. The headache was without pain dissemination, feelings of heart palpitation, or nausea. She took 100 mg diclofenac suppository every day, but no improvement was observed in symptoms. No seizure or loss of consciousness had been reported. She felt fatigue as her sleep duration increased and her daily activities slowed down. At the same time, chickenpox lesions appeared on the surface of the skin and low-grade fever continued during those 11 days.

The patient was married 5 years ago and has a 5-year-old child. In her history, she has had only one pregnancy without any abortion or stillbirth. Her menstruation period was regular, and the volume of bleeding was within the normal range.

Before her pregnancy, a 3-cm myoma had been diagnosed by ultrasound. Owing to the breech position of the fetus and gradual increase in myoma size up to 30 cm, cesarean section was selected by horizontal incision. Laparoscopic myomectomy was performed 1 year after delivery, and owing to abdominal pain and discomfort, endometriosis was diagnosed with computed tomography (CT) scan at the site of the cesarean section about 3 years later. Continuous use of Diane-35 for 3 months was advised by her physician. Diane is a combination of cyproterone and ethinyl estradiol. At the onset of the headache, she had been taking the Diane tablet for 45 days. At the time, she had been taking painkiller drugs and the Diane tablet. She has no past systemic medical history or positive family history for neurological and gynecological problems.

**Fig. 1:**
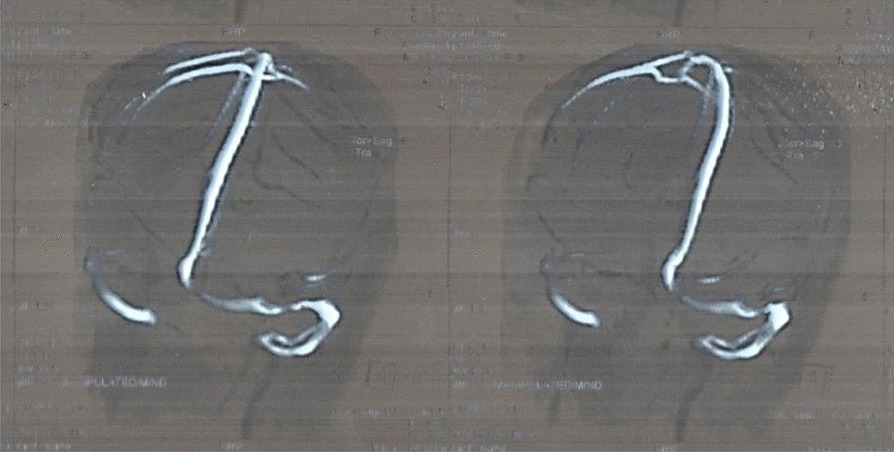
Slight irregularity and narrowing on the right transverse sinus, which may have been caused by the previous CVT

She was hospitalized. The necessary physical and laboratory examinations were performed, and the results were as follows: Glasgow score (GCS) was 15/15, cranial and funduscopic examinations were normal, no double vision had occurred, and Kernig’s sign, Brudzinski’s sign, and neck redor were negative. A CT scan was performed, and suspicion of vein involvement was reported. After that, the patient underwent MRV, which revealed irregularity and occlusion in the veins of the right sinus transverse and posterior part of the superior sagittal sinus were reported (Fig. 1).

Pantoprazole 40 mg twice a day, enoxaparin 60 U subcutaneously twice a day, dexamethasone Ampoule (Amp) 4 mg three times a day, acetazolamide tablet 250 mg three times a day, gabapentin capsule 100 mg every night, and warfarin tablet 5 mg per day were ordered. A review of hematology and rheumatology findings is presented in Tables 1 and 2. Owing to the lack of access to genetic testing for procoagulative disorders, these tests were not performed. She was hospitalized for 15 days in the general ward of the hospital. After discharge, half of warfarin tablet 5 mg per day and acetazolamide 250 mg was administered three times a day with close and careful follow-up of the International Normalized Ratio (INR). Thirty-two days after cerebrovascular thrombosis, another MRV was taken and there was no evidence of sinus thrombosis. After 8 months of taking warfarin, the drug was changed to clopidogrel (Zyllt^R^). Two years later, the physician discontinued the antiplatelet drugs, but after a month the headache returned. On retaking clopidogrel, she became symptom free.

**Table 1:** Lab test findings of patient

Lab test	Result	Normal range
CBC		
WBC (×10^3^/µL)	6.5	4.0–11.0
MCHC (g/dL)	31	31.0–36.0
MCH (pg)	20.3	28.0–32.0
MCV (µm^3^)	65.5	78–96
Hb (g/dL)	8.7	13–18
Hct (%)	28.1	38.8–50
RBC (×10^6^/µL)	4.29	4.00–6.00
Plt (×10^3^/µL)	297	150–400
Lym (%)	40	20–40
Neut (%)	50	55–73
ESR (mm/hour)	20	0–29
CRP	Neg	
TPI	Neg	
Mg (mg/dL)	1.9	1.5–2.5
Alp (U/L)	141	44–147
Bili		
Total (mg/dL)	0.3	0.2–1.2
Direct (mg/dL)	0.1	0–0.4
Fe (µg/dL)	77	60–170
P (mg/dL)	3	2.5–4.5
Ca (mg/dL)	9.1	8.5–10.2
Na (mEq/L)	140	135–145
K (mEq/L)	3.7	3.5–5.0
Cr (mg/dL)	0.8	0.5–1.1
Urea (mg/dL)	21	7–20
BS (mg/dL)	80	72–99

*CBC* complete blood count, *WBC* white blood cell, *MCHC* mean corpuscular hemoglobin concentration, *MCH* mean corpuscular hemoglobin, *MCV* mean corpuscular volume, *Hb* hemoglobin, *Hct* hematocrit, *RBC* red blood cell, *Plt* platelet, *Lym* lymphocyte, *Neut* neutrophil, *ESR* erythrocyte sedimentation rate, *CRP* C-reactive protein, *TPI* troponin T, *Mg* magnesium, *Alp* alkaline phosphatase, *Bili* Bilirubin, *Fe* iron, *P* phosphorus, *Ca* calcium, *Na* sodium, *K* potassium, *Cr* creatinine, *BS* blood sugar

**Table 2:** Rheumatologic lab tests of patient

Rheumatologic lab tests	Result	Normal range
ANA	0.15	<3
Anti-dsDNA Ab	23.4	>10
Anti-phospholipid IgG	2.7	<15
Anti-phospholipid IgM	1.7	<12
Lupus anticoagulant	Neg	
C3	135.2	80–160
C4	25.8	16–48

*ANA* antinuclear antibody, *Anti-dsDNA Ab* anti-double-stranded DNA, *IgG* immunoglobulin G, *IgM* immunoglobulin M, *C3* complement component 3, *C4* complement component 4

## Discussion

We reported a woman who took OCPs for a short period of time and showed clinical symptoms of CVT. Superior sagittal sinus is the most common sinus that is involved in CVT [2, 8]. CVT symptoms are classified from mild to severe, so in some patients a headache is the only main complaint [3]. Endometriosis is an estrogen-dependent disease, so contraceptive pills are considered the first line of treatment [9] Current studies have showed that women with endometriosis appeared to be in a state of hypercoagulability, and this coagulation dysfunction potentially contributed to the inflammatory nature of the ectopic lesions. It has been estimated that 18% of women in developing countries use OCPs [10]. Contraceptive pills can alter the results of prothrombotic tests and increase coagulopathies [11]. Also, OCPs can increase the risk of cardiovascular diseases, CVT, breast cancer, and melanoma [12, 13].

If neurological disorders are suspected in women of reproductive age, their medical history should be examined for use of OCPs [10]. Diane pill is a combination of estrogen and progesterone and is known as a combined oral contraceptive (COC). Our patient had taken it for 45 days. Dehydration and fasting increased the odds ratio of CVT [1]. Magnetic resonance imaging (MRI) is known as an available method for easy detection of CVT [3]. Also, CVT can be fatal owing to epilepsy status, infection, and herniation [3]. Three main treatments of CVT are the removal of etiologic factors, anticoagulants administration, and symptoms treatment [3]. A previous study suggested that the effective course of anticoagulant therapy is maximum 12 months [2].

Our patient underwent anticoagulant therapy because of discontinuation of clopidogrel, and the symptoms of headache returned. Endometriosis is the abnormal growth of endometriosis tissue and its ectopic implantation [9]. Ten percent of women of reproductive age suffer from endometriosis [9, 14]. Inflammatory agent and estrogen are two effective factors in endometriosis [9]. Symptoms of clinical endometriosis include dysmenorrhea, pelvic pain, chronic fatigue, and abnormal uterine bleeding, but some patients have no symptoms [14]. The main strategy of treatment is to suppress ovarian function [14]. During 6 months of COC use, danazol, gestrinone, medroxyprogesterone acetate, and gonadotropin-releasing hormone (GnRH) agonists are intended for patients [14]. In patients with a history of OCP use, thrombosis events should be considered [15]. Dehydration and OCPs together increase the risk of CVT [1]. Our patient suffered from chickenpox, and dehydration could increase OCP concentration.

## Conclusion

The evidence from this study suggests that, although CVT symptoms are variable, new presentation of headache complaints can be considered one of the early symptoms of CVT. We think there is a significant relationship between past history of OCP use in women of reproductive age that should be considered as an important diagnostic factor as well. Finally, MRV should be utilized as an accurate diagnostic tool.

## Abbreviations

CVT: Cerebral vein thrombosis
OCPs: Oral contraceptive pills
GCS: Glasgow score
MRV: Magnetic resonance venography
INR: International normalized ratio
COC: Combined oral contraceptive
MRI: Magnetic resonance imaging
GnRH: Gonadotropin-releasing hormone
